# Environment-Stable Co_*x*_Ni_*y*_ Encapsulation in Stacked Porous Carbon Nanosheets for Enhanced Microwave Absorption

**DOI:** 10.1007/s40820-020-00432-2

**Published:** 2020-04-28

**Authors:** Xiaohui Liang, Zengming Man, Bin Quan, Jing Zheng, Weihua Gu, Zhu Zhang, Guangbin Ji

**Affiliations:** 1 College of Materials Science and Technology, Nanjing University of Aeronautics and Astronautics, Nanjing, 211100 People’s Republic of China; 2 Department of Chemistry and Materials Science, College of Science, Nanjing Forestry University, Nanjing, 210037 People’s Republic of China

**Keywords:** MOFs, Co_*x*_Ni_*y*_@C nanosheets, Multiple interfaces, Microwave absorption, Environmental stability

## Abstract

Magnetic/dielectric@porous carbon composites, derived from metal–organic frameworks (MOFs) with adjustable composition ratio, have attracted wide attention due to their unique magnetoelectric properties. In addition, MOFs-derived porous carbon-based materials can meet the needs of lightweight feature. This paper reports a simple process for synthesizing stacked Co_*x*_Ni_*y*_@C nanosheets derived from Co_*x*_Ni_*y*_-MOFs nanosheets with multiple interfaces, which is good to the microwave response. The Co_*x*_Ni_*y*_@C with controllable composition can be obtained by adjusting the ratio of Co^2+^ and Ni^2+^. It is supposed that the increased Co content is benefit to the dielectric and magnetic loss. Additionally, the bandwidth of CoNi@C nanosheets can take up almost the whole Ku band. Moreover, this composite has better environmental stability in air, which characteristic provides a sustainable potential for the practical application.

**Electronic supplementary material:**

The online version of this article (10.1007/s40820-020-00432-2) contains supplementary material, which is available to authorized users.

## Highlights

- The microwave absorbing performance of alloy@C composites can be controlled through regulating ratio of metal ions.
- Carbon-based alloy@C composites exhibit the potential stability of microwave absorption with almost the whole Ku band for the practical application.

**Electronic supplementary material:**

The online version of this article (10.1007/s40820-020-00432-2) contains supplementary material, which is available to authorized users.

## Graphical abstract

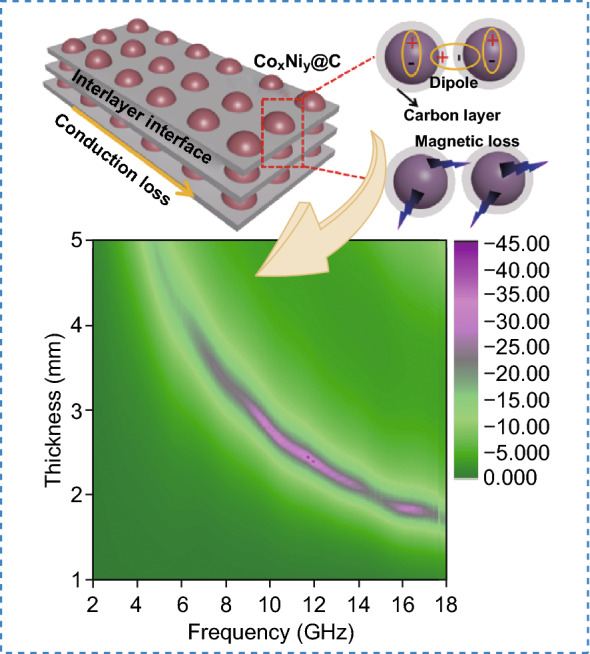

## Introduction

With the rapid expansion of communication technology and increasing electromagnetic radiation, it is necessary to achieve multifunctional absorbers [1–3]. Various strict performance requirements such as thin thickness, light weight, wide frequency band, and strong absorption strength have been proposed [4, 5]. Hence, the study of the composition and structure design of the material has been stimulated [6–8]. Among them, magnetic/dielectric [9–11] composites have received more attention due to the excellent dielectric and magnetic losses. For example, Zhou et al. [12] reported a non-uniform FeCo/ZnO nanosheet that was adjusted by an auxiliary template method to reduce the density and impedance of the composite. By adjusting Ni^2+^ artificially designed Co_*x*_Ni_*y*_@C structure, a strong electromagnetic wave response was obtained by Quan et al. [13]. Che et al. [14] reported CoNi@Air@TiO_2_ yolk-shell structure with outstanding microwave absorption property (RL = − 58.2 dB). Feng et al. [15] also investigated the CoNi alloy combined with TiO_2_ and graphene, and the matching thickness is only 2.0 mm. All of them demonstrated the strong magnetic loss caused by CoNi cores. Therefore, the CoNi alloy could be a candidate for the magnetic loss material.

In addition, the ideal absorber should have the characteristics as follows: strong magnetic lossand sufficient dielectric loss [16, 17]. Porous carbon is considered to be a material with high dielectric loss [18, 19]. Moreover, due to the lightweight property of porous carbon, assembling alloy in carbon materials is a commendable choice. However, the problem is that the process of preparing alloy@porous carbon materials by the conventional template method is complicated [20, 21]. Therefore, with a sample method to prepare alloy nanoparticles embedded in porous carbon is a challenge. Metal/oxide nanoporous carbon composites derived from MOFs have an easy-to-access surface area, diverse structural topologies, and adjustable functions, which is a mature synthesis method developed in recent years [22, 23].

In this study, stacked CoNi-MOFs used as a template deriving Co_*x*_Ni_*y*_@C nanosheets have been investigated. It is worth noting that the carbonization process is important for the formation of porous carbon and Co_*x*_Ni_*y*_ alloys and the stacked structures promote the formation of multiple interfaces. The synthesized Co_*x*_Ni_*y*_@C composite has a highly developed porous structure. In the derived porous structure, the carbon layer can protect the metal molecules from oxidation [24]. Moreover, the carbon layer can provide a channel for electron transport, which is good for dielectric loss [25, 26]. In addition, for the CoNi@C(Co^2+^: Ni^2+^= 1:1) nanosheets, the maximum reflection loss value is − 43.7 dB, and the lower thickness is 1.7 mm with the filler loading ratio of 20 wt%. In addition, the effective bandwidth is reaching 5.7 GHz with thinner thickness of 1.8 mm. This study has shown that Co_*x*_Ni_*y*_@C nanosheets are excellent adsorbents because of their light weight, thin thickness, and strong absorption capacity. At the same time, this research also opened up a new way for simply designing multiple interfaces and stable porous nanostructured alloy@carbon nanosheets with targeted functions.

## Experimental Section

### Synthesis of Co_*x*_Ni_*y*_@C Nanocomposites

CoNi-MOF:60 mL of DMF (dimethylformamide) dissolved 438 mgCo(NO_3_)_2_·6H_2_O and 436 mg Co(NO_3_)_2_·6H_2_O (molar ratio = 1:1), 633 mg H_3_BTC (1,3,5-benzenetricarboxylic acid) and 576 mg 4,4′-bipyridine. The supernatant was stirred vigorously for 30 min and then transferred to a Teflon-lined stainless steel autoclave heating at 120 °C for 4 h. Finally, the resulting powder was centrifuged and washed vigorously with DMF and absolute ethanol. The clean powder was dried under vacuum at 80 °C for 12 h. For the Co_3_Ni_7_-MOF and Co_7_Ni_3_-MOF, the molar ratio of Co^2+^ and Ni^2+^ is 3:7 and 7:3, respectively, and other conditions are same as the CoNi-MOF. Then, the Co_*x*_Ni_*y*_-MOF was directly calcinated at 800 °C with heating rate of 2 °C min^−1^ for 2 h to obtain the Co_*x*_Ni_*y*_@C composites under nitrogen atmosphere. In addition, the CoNi@C composites were placed in a sample box covered with a breathable plastic film and left it for 1 year in the natural environment, which is named as CoNi@C-1. Moreover, CoNi-MOFs calcined at 700, 800, and 900 °C with heating rate of 2 °C min^−1^ were named S-700, S-800, and S-900, respectively.

### Structure Characterization

FESEM (field-emission scanning electron microscopy, JEOL, JSM-7100F) and TEM (transmission electron microscopy, JEOL, JEM-2100F) were used to analyze the morphology and microstructure of the Co_*x*_Ni_*y*_@C nanosheets. Raman spectra (Renishaw INVIA micro-Raman spectroscopy system) and XRD (D8 Advance X-ray diffractometer, Cu K*α* radiation, *λ* = 1.5418 Å) was used to characterize the structure of the Co_*x*_Ni_*y*_@C nanosheets. X-ray photoelectron spectroscopy (XPS, VGMultiLab 2000) was used to test the chemical states of elements. Adsorption of nitrogen was used to measure Brunauer–Emmett–Teller (BET) surface area using Tristar II 3020 instrument. Agilent PNA N5244A vector network analyzer (VNA) was used to test the electromagnetic parameters in the range of 2–18 GHz with coaxial wire analysis model [27]. Compressing sample and paraffin with 20% filler loading ratio made a ring with inner and outer diameter of 3.04 mm and 7.00 mm to measure.

## Results and Discussion

### Structure of Co_*x*_Ni_*y*_-MOFs and Co_*x*_Ni_*y*_@C Composites

**Fig. 1:**
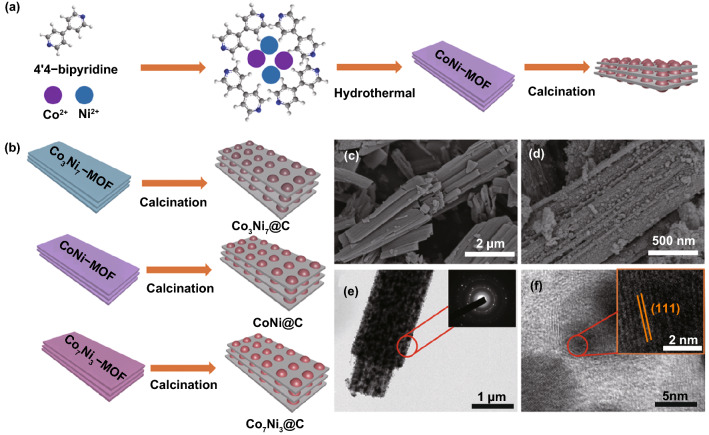
**a**, **b** Schematic diagram process for forming of Co_*x*_Ni_*y*_@C. **c** SEM picture of CoNi-MOF. **d** SEM picture of Co_3_Ni_7_@C nanosheets. **e**, **f** TEM and HRTEM of Co_3_Ni_7_@C nanosheets

In order to comprehend the formation process of Co_*x*_Ni_*y*_@C clearly, typical synthesis route is shown in Fig. 1a, b. Stacked precursors with nanosheets morphology were synthesized firstly by hydrothermal method. Then, the precursors were placed in a railboat annealing in the N_2_ atmosphere at 800 °C obtained the Co_*x*_Ni_*y*_@C nanosheets. In fact, the carbonization process is important for the formation of the Co_*x*_Ni_*y*_ alloy and the formation of a porous carbon skeleton, and the stacked nanosheets formed multiple interfaces attenuating microwave. During the calcination process, a partially graphitized carbon layer covered the Co^2+^ and Ni^2+^. At the same time, carbon reduces the metal ions Ni^2+^ and Co^2+^ to Ni^0^ and Co^0^ and then melts them into a Co_*x*_Ni_*y*_ alloy according to the feed ratio. Finally, the derived Co_*x*_Ni_*y*_ alloy nanoparticles were implanted in carbon layer to obtain Co_*x*_Ni_*y*_/C nanosheets.

Figure 1c, d shows the SEM pictures of CoNi-MOF precursor and CoNi@C nanosheets. Figure 1c exhibits relatively smooth stacked nanosheets morphology with a breadth about 1 μm, and Fig. S1a shows the porous cross profile of the CoNi-MOF precursor. After calcination, the stacked structure became loose and the primary smooth surface became rough (Fig. 1d), emerging more porous structure. In addition, each contact layer forms an interface. Figure S1b, c shows the morphology of Co_3_Ni_7_@C and Co_7_Ni_3_@C composites, the morphology of Co_3_Ni_7_-MOF and Co_7_Ni_3_-MOF is inserted, respectively. All of the Co_*x*_Ni_*y*_@C exhibited loose porous structure, which is good to microwave absorption. Figure 1e, f show TEM and HRTEM images of CoNi@C composite, respectively. It can be clearly seen that the nanosheets are stacked. In addition, the 0.206 nm lattice fringes (obtained from red area in Fig. 1f) can be observed clearly, which is corresponded to (111) plane spacing of the face-centered cubic of CoNi crystal [13]. At the edge of CoNi alloy, there is the lattice fringe of the amorphous carbon indicating the presence of carbon layer at the outside of the CoNi alloy. Figure 1e presents the CoNi@C alloy nanoparticles are equably dispersed on the carbon nanosheets, and the selected area electron diffraction insert in Fig. 1e demonstrated the polycrystalline property of the CoNi@C composites. The energy-dispersive X-ray elemental mappings of Co_*x*_Ni_*y*_@C are displayed in Fig. S1di, showing the distribution of Co and Ni elements. Figures S1d, e show the Co_3_Ni_7_@C nanosheets elements mapping, which indicated the content of Co is lower than Ni. Figures S1f, g show the CoNi@C nanosheets elements mapping, which illustrated the content of Co is nearly to Ni. Figures S1h, i show the Co_3_Ni_7_@C nanosheets elements mapping, which stated the content of Co is more than Ni. All of results are corresponding to the synthesis progress. In addition, according to the TG analysis in Fig. S2a, the carbon contents in Co_3_Ni_7_@C, CoNi@C, and Co_7_Ni_3_@C are evaluated to be 8.83%, 9.82%, and 10.28%, respectively. It could be concluded that the weight loss from 0 to 100 °C is water, and the weight loss from 100 to 1000 °C is carbon in the Co_*x*_Ni_*y*_@C composite [28].

**Fig. 2:**
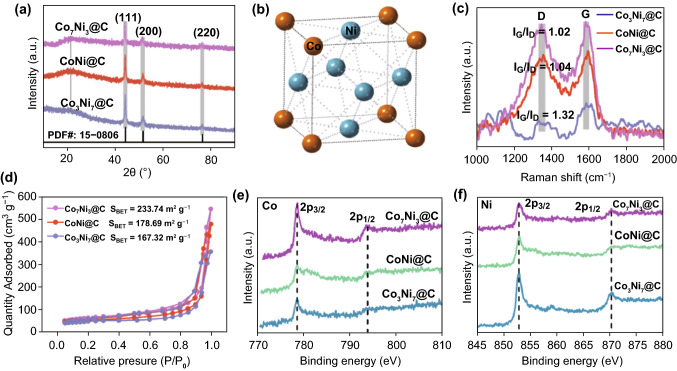
**a** XRD pattern of Co_*x*_Ni_*y*_@C composites. **b** Atomic structure diagrams of CoNi. **c** Raman spectra of Co_*x*_Ni_*y*_@C composites. **d** Nitrogen adsorption–desorption isothermals of Co_*x*_Ni_*y*_@C composites. XPS spectra of CoNi@C **e** Co 2*p* and **f** Ni 2*p*

The successful synthesis could be proven by XRD pattern of the obtained Co_*x*_Ni_*y*_@C composites (Fig. 2a). The (111), (200), and (220) faces peaks of face-centered cubic (*fcc*) Co_*x*_Ni_*y*_ alloy are matched between 40° and 80° [13], in which the fcc atomic structure diagrams can be seen from Fig. 2b. Additionally, all peak locations are resembled to *fcc*  Ni (JCDPS No. 15-0806) or *fcc* Co (JCDPS No. 01-1260) [13]. Furthermore, no other impurity peak was found, indicating that only the pure Co_*x*_Ni_*y*_ alloys were synthesized. In addition, it can be known from the Raman in Fig. 2c, there are carbon layers in the composites. In general, the D-band is relative to the local defects and disorders carbon [29]. The G-band at 1587 cm^−1^ is supported to the *E*_2g_ phonon of *sp*^2^ bonds of carbon atoms, corresponded to the carbon graphitization degree [30]. As shown in Fig. 2c, with the increase in Co content, the G-band shows higher values, especially for the Co_7_Ni_3_@C composites, which indicated existing much graphitic carbon nanostructure in the Co_7_Ni_3_@C nanosheets. It is because the Co metal could catalyze the formation of graphitic carbon [31]. Additionally, in order to illustrate the influence of heating treatment temperature on CoNi@C composites, the Raman spectra of CoNi@C is shown in Fig. S2b. It also demonstrated that the *I*_G_/*I*_D_ values were increased with the high temperature. It can be inferred that the calcination temperature is conducive to the degree of graphitization of carbon, which will tune the electromagnetic wave loss capability [32]. However, the much graphitic carbon may result in higher conductivity, which prejudice against microwave absorption. In addition, the Co_*x*_Ni_*y*_@C nanosheets also possess the nanoporous structures. In Fig. 2d, the specific surface areas of all the composites were tested. The BET surface areas of Co_3_Ni_7_@C, CoNi@C, and Co_7_Ni_3_@C composites are 167.32, 178.69, and 223.74 m^2^ g^−1^, respectively, which become larger as the Co content increases. From the pores size distribution shown in Fig. S2c, it can be seen the Co_*x*_Ni_*y*_@C nanosheets appear nanoporous structure, which could provide more contact site for microwave attenuation. The enhanced specific surface areas illustrate the presence of more pore structures with the increasing Co content. Moreover, XPS was used to further analyze the chemical valences and elemental composition. XPS survey spectrum of the CoNi@C nanosheets is presented in Fig. S3a. Obvious peaks of C, Co, and Ni elements were obtained. In addition, the intensity of Co and Ni peaks increases with the increase in Co^2+^ and Ni^2+^ concentration. It is obviously noted that the Co 2*p* (Fig. 2e) and Ni 2*p* (Fig. 2f) were all observed in the three samples; moreover, the Co 2*p* and Ni 2*p* peaks of CoNi@C in Fig. S3b, c demonstrated the presence of Co and Ni metal [33], which also indicated the successful synthesis of Co_*x*_Ni_*y*_ alloy. The C 1*s* in Fig. S3d could also illustrate the formation of C.

### Microwave absorption

**Fig. 3:**
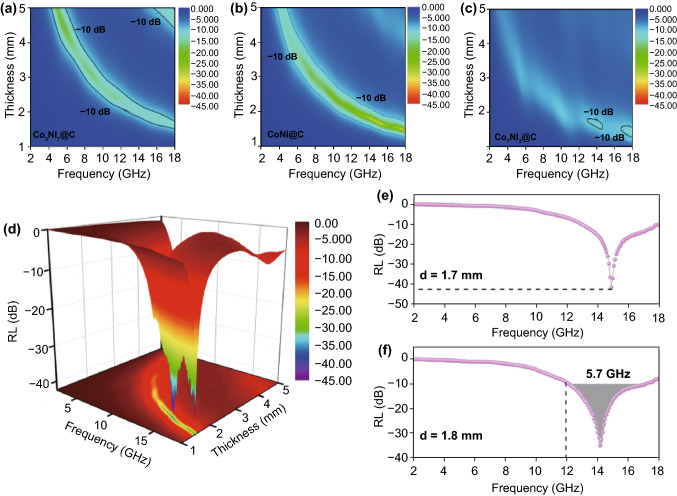
Reflection loss contour map of **a** Co_3_Ni_7_@C, **b** CoNi@C, and **c** Co_7_Ni_3_@C nanosheets. **d** 3D RL plots. RL plots with **e** 1.7 mm and **f** 1.8 mm thickness of CoNi@C nanosheets

In order to explore the electromagnetic wave absorption performance of the composites, reflection loss (RL) values of all samples with 20 wt% filler loading ratios are displayed in Fig. 3a–c. Based on Eqs. 1 and 2 [34, 35]:

1 $${\text{RL}} = 20 \log \left| {\frac{{Z_{\text{in}} - Z_{0} }}{{Z_{\text{in}} + Z_{0} }}} \right|$$

2 $$Z_{\text{in}} = Z_{0} \sqrt {\frac{{\mu_{r} }}{{\varepsilon_{r} }}} \tanh \left( {j\frac{2\pi fd}{c}\sqrt {\frac{{\mu_{r} }}{{\varepsilon_{r} }}} } \right)$$

where *Z*_in_ and *Z*_0_ are the input impedance of the absorber and impedance of air. *ε*_*r*_ and *μ*_*r*_ are normalized complex permittivity and permeability of the absorber. *f*, *d,* and *c* represent the frequency of incident microwaves, the thickness of absorber, and the velocity of light, respectively. In general, the RL value below − 10 dB indicates that 90% of the microwave is absorbed and it can be considered as an effective absorption. However, in practical applications, there is a strong requirement for wide bandwidth and thin matching thickness. From reflection loss contour map at different thickness in Fig. 3a–c, it can be seen compared with Co_3_Ni_7_@C and Co_7_Ni_3_@C composites, and CoNi@C nanosheets could be obtained evident better microwave absorption performance with below 2 mm thickness. In addition, the 3D reflection loss map could more visually present the RL values of the composites. Compared with RL values of Co_3_Ni_7_@C (Fig. S4a) and Co_7_Ni_3_@C (Fig. S4b) composites, the CoNi@C sample (Fig. 3d) exhibited the strongest microwave absorption performance with − 43.7 dB at 1.7 mm thickness (Fig. 3e). Furthermore, the effective absorption bandwidth 5.7 GHz of CoNi@C nanosheets in almost whole Ku band could be obtained only with thicknesses of 1.8 mm (Fig. 3f).

In order to verify the fact that the CoNi@C composites obtained at 800 °C possess the best microwave absorption performance, the electromagnetic characteristics of CoNi@C composites with different calcination temperatures are compared in Fig. S5. It can be seen that as the calcination temperature increases, the dielectric constant gradually increases (Fig. S5a, b), and the variety in magnetic permeability (Fig. S5c) is not obvious. The increase in the dielectric constant indicates that the dielectric attenuation characteristics of the composite are enhanced, which is conducive to electromagnetic wave absorption. However, through the comparison of RL, it was found that the reflectance of S-700 (Fig. S5d) and S-900 (Fig. S5e) samples did not reach − 10 dB, and S-800 (Fig. 3e) showed excellent reflection loss.

**Fig. 4:**
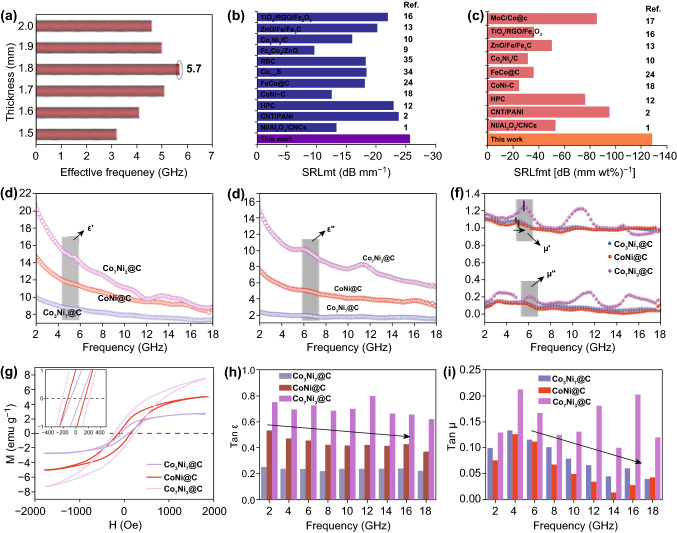
**a** Effective frequency bandwidth of CoNi@C. **b** SRL_mt_ and **c** SRL_fmt_ of carbon-based materials. **d** Real part and **e** imaginary part of permittivity of Co_*x*_Ni_*y*_@C nanosheets. **f** Permeability of Co_*x*_Ni_*y*_@C nanosheets. **g** Magnetic hysteresis loops of Co_*x*_Ni_*y*_@C nanosheets. **h** Dielectric loss tangent and **i** magnetic loss tangent of Co_*x*_Ni_*y*_@C nanosheets

To explore the maximum absorption bandwidth below 2 mm thickness, the effective frequency bandwidth of CoNi@C composites with 1.5-2 mm is shown in Fig. 4a. By comparison, the broadest absorption bandwidth could only be acquired at 1.8 mm thickness. Another method to evaluate the microwave absorption property is performed in Fig. 4b, c and Table S1. SRL_mt_ (RL/matching thickness) (Fig. 4b) and SRL_fmt_ (RL/(filler loading × matching thickness)) of CoNi@C nanosheets were calculated with comparing these values with reported carbon-based nanosheets materials. Obviously, the much higher SRL_mt_ and SRL_fmt_ values of CoNi@C composites outclass the reported composites, which implied the better prospect for CoNi@C as an ultrathin, ultralight, and highly effective microwave absorber. In order to clear the cause of microwave absorption gap with three different samples, the electromagnetic parameters are analyzed in Fig. 4d-f. The values of *ε*′ (Fig. 4d) and *ε*″ (Fig. 4e) decreased in the 2–18 GHz range, which exhibited frequency dispersion effect benefited to incident microwave dissipation, conductivity, and dielectric loss. The *ε*′ and *ε*″ are increased with the added content of Co, which also illustrate the catalytic effect on graphitized carbon [30]. Although the decline of the *ε*″ is not good to the dielectric loss, the tangent (tan*δ*_*ε*_ = *ε″/ε′*) [36] (Fig. 4h) illustrate that the dielectric loss was increased with the addition of Co. At the same time, analogical trends also emerged in complex permeability (*μ*′ and *μ*″ in Fig. 4f), which indicated outstanding magnetic loss behavior. The magnetic losses are usually associated with natural resonance, exchange resonance, and eddy current loss [37].The eddy current loss is determined by *C*_0_ (*C*_0_ = *μ′*(*μ″*)^−2^*f*^−1^ = 2*Πμ*_0_*d*^2^*σ/*3) [38], if the main reason for magnetic loss is the eddy current loss, the *C*_0_ values are constant. It is obvious that the *C*_0_ value fluctuates and decreases in 2–18 GHz frequency range (Fig. S6b). Therefore, eddy current loss is not the dominant mechanism of magnetic loss, so the exchange resonance and natural resonance should be noticed. The natural resonance usually takes place from 0.1 to 10 GHz [39]. Hence, the peaks of *μ*″ at 6 GHz (Fig. 4f) are related to the natural resonance. The two peaks of *μ*″ at 11.5 and 15 GHz (Fig. 4f) are relevant to exchange resonance. In addition, the natural resonance and exchange resonance are all enhanced due to the improved magnetism by Co, in spite of the only the enhancement of Co_7_Ni_3_@C complex is more obvious. Although the natural and the exchange resonance processes cause a decrease of *µ*’ and *μ*″, the magnetic loss still was enhanced with the increase in Co content in Co_*x*_Ni_*y*_@C composites. The M-H loop [40] of Co_*x*_Ni_*y*_@C nanosheets variation up with increased content of Co is shown in Fig. 4g to further prove the increased magnetic property. The saturation magnetization value (Fig. 4g) was much lower than pure Co_*x*_Ni_*y*_ alloy, which is because the dielectric carbon layer wrapped outside of the Co_*x*_Ni_*y*_ alloy. It is general to know the magnetic tangent (tan*δ*_*μ*_ = *μ″/μ′*) [36] are dominant criterion for evaluating magnetic loss. With the increased content of Co, themagnetic loss (Fig. 4i) was increased. Therefore, the increase of Co content improved dielectric and magnetic loss at the same time, which also enhanced the impedance matching (*Z*_*r*_ = *Z*_in_*/Z*_0_). *Z*_*r*_ value close to 1 indicates better impedance matching. From Fig. S6a, one can find that the CoNi@C composites achieved the best impedance matching. Therefore, it illustrates that appropriate ratio of Co and Ni content was good for dielectric loss, while a superfluous was against the impedance matching, resulting in the miserable microwave absorbing performance.

**Fig. 5:**
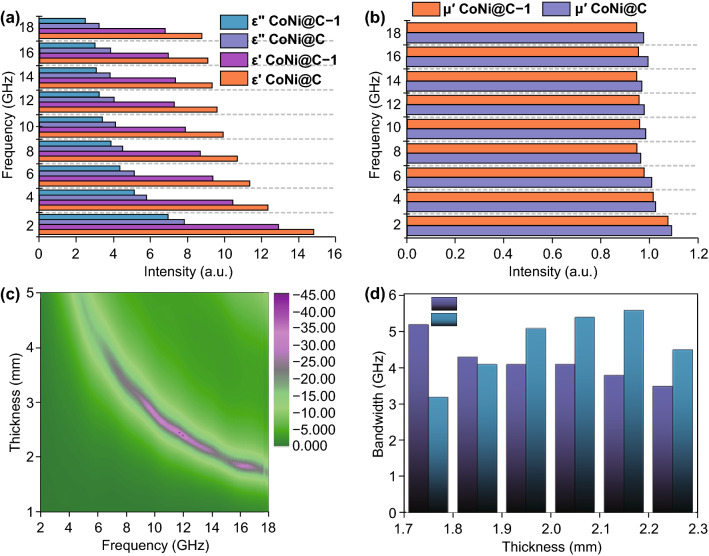
**a** Permittivity and **b** real part of permeability of CoNi@C composites and after 1 year of exposure in air.**c** RL values of CoNi@C composites exposure in air after a year. **d** Effective bandwidth of CoNi@C and after a year of exposure in air with different thicknesses

Furthermore, in order to illustrate the microwave absorption stability of the Co_*x*_Ni_*y*_@C composites, the CoNi@C samples as representative were exposed in air for 1 year later to test the microwave absorption.All the measured values of *ε′*, *ε*″, and *μ′* of CoNi@C and CoNi@C-1(Fig. 5a, b) shown declined a little bit after exposing in air for 1 year later. Additionally, the RL values (Fig. 5c) and effective bandwidth (Fig. 5d) of CoNi@C-1 composites further proved the stability. Although the decline of the permittivity and permeability unavoidably weakens the attenuation ability for microwave, the CoNi@C-1 composites still appeared better RL loss of − 35 dB with 1.85 mm thickness (Fig. 5c). Broadband effective absorption bandwidths could be successfully reached 5.1 GHz with thickness of 2.15 mm. Therefore, the CoNi@C composites can keep better stability in air for 1 year or longer time with strong microwave response.

**Fig. 6:**
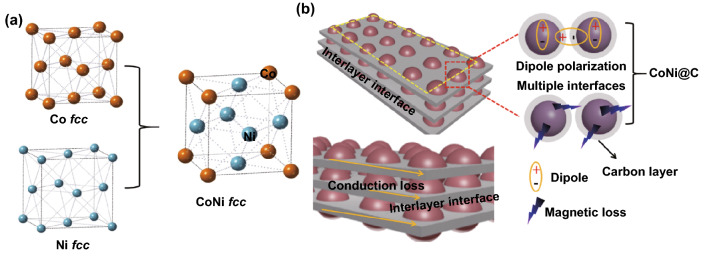
**a** Atomic structure diagrams of Co, Ni, and CoNi. **b** Possible microwave absorption mechanism of Co_*x*_Ni_*y*_@C

In addition to the mechanism of microwave attenuation described above, the conduction loss is another important factor to consume electromagnetic energy. Figure 6a presents atomic structure diagrams of the fcc Co and fcc Ni forming fcc CoNi alloy, which structure increased the stability of the CoNi alloy particles. Moreover, the Co amount affecting the dielectric properties is also proved by density functional theory (DFT) calculations [41]. Because of the increased Co content, the strong conductive loss was good to microwave attenuation.The mechanism of electromagnetic energy conversion in this study can be well revealed, based on the original work reported by Cao and his co-workers that electron transport and dipole polarization do competitive synergy on electromagnetic attenuation [7]. In Fig. 6b, the mechanism of microwave absorption is presented comprehensively, including electron transmission conduction loss, stacked porous nanosheets providing more contact site for microwave, dipole polarization between the CoNi alloy and carbon layer, and dielectric and magnetic loss. Among them, electron transmission conduction loss mainly come from the carbon nanosheets, and the modes of electron transmission could be explained by Yuan et al. [42, 43]. Both electron transport and dipole polarization have great impact on high-performance electromagnetic attenuation, which can be well explained by their competitive synergy originally reported by Cao et al. [44, 45]. Additionally, the stacked nanosheets could also form interlayer interfaces and the Co_*x*_Ni_*y*_@C nanoparticles could provide multiple interfaces, when electromagnetic waves enter different interfaces, the attenuation degree of loss is different; therefore, multiple interfaces allow electromagnetic waves to be attenuated to a greater extent.

## Conclusion

In summary, the stacked Co_*x*_Ni_*y*_@C nanosheets were successfully synthesized by adding CoNi-MOF derived changing with Co^2+^ and Ni^2+^. The microwave absorption loss mechanism included interfaces attenuation brought by stacked structure, conduction loss induced by electron transport, dielectric loss created by carbon, magnetic loss, natural and exchange resonance caused by Co_*x*_Ni_*y*_ alloy, and dipole polarization brought by defective carbon and Co_*x*_Ni_*y*_@C nanoparticles. Microwave absorption performance with a minimum RL value of − 43.7 dB with 1.7 mm thin thickness and an effective absorption bandwidth of 5.7 GHz with 1.8 mm thickness could be achieved with a lower filler loading ratio of 20 wt%. Benefiting from the abrasive porous nanosheets structure, it can provide more exposure site for microwave scattering. Therefore, stacked CoNi-MOF-derived multiple interfaces Co_*x*_Ni_*y*_@C nanosheets provided new ideas for the synthesis of alloy@C composites and increase applications in the microwave absorption field.

## Supplementary Material

SEM, TG, Raman, pore size distribution, XPS, RL permittivity, permeability, *Z*_*r*_, and *C*_0_ curves of the composites, microwave absorption property comparisons of reported literature and this work.

## Electronic supplementary material

Below is the link to the electronic supplementary material.

Supplementary material 1 (PDF 717 kb)
