# Supplementary material for: Environment-Stable CoxNiy Encapsulation in Stacked Porous Carbon Nanosheets for Enhanced Microwave Absorption
Source: Nanomicro Lett. 2020 Apr 28;12:102. doi: 10.1007/s40820-020-00432-2 (PMC7770751; doi:10.1007/s40820-020-00432-2)
Supplement: Supplementary file 1 — Supplementary material 1 (PDF 717 kb) [file 40820_2020_432_MOESM1_ESM.pdf]

Supporting Information for

Environment-stable Co_xNi_y Encapsulation in Stacked Porous Carbon Nanosheets for Enhanced Microwave Absorption

Xiaohui Liang[1], Zengming Man[1], Bin Quan[1], Jing Zheng[2], Weihua Gu[1], Zhu Zhang[1], Guangbin Ji[1, *]

[1]College of Materials Science and Technology, Nanjing University of Aeronautics and Astronautics, Nanjing 211100, People's Republic of China

[2]Department of Chemistry and Materials Science, College of Science, Nanjing Forestry University, Nanjing 210037, People's Republic of China

*Corresponding author. E-mail: gbji@nuaa.edu.cn (Guangbin Ji)

Supplementary Figures and Table

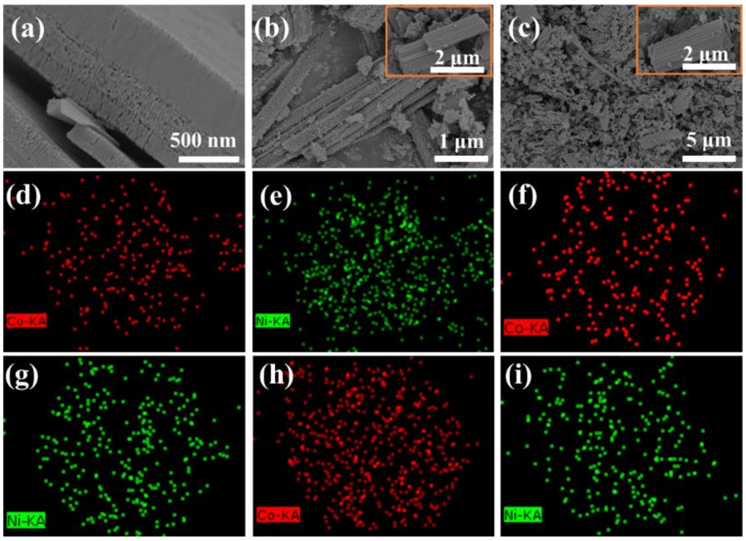

Fig. S1 a SEM cross profile picture of CoNi-MOF. SEM picture of **b** Co_3Ni_7@C nanosheets and the insert SEM of the Co_3Ni_7-MOF and **c** Co_7Ni_3@C nanosheets and the insert SEM of the Co_7Ni_3-MOF. **d-e** elements mapping of Co_3Ni_7@C, **f-g** CoNi@C, **h-i** Co_7Ni_3@C nanosheets

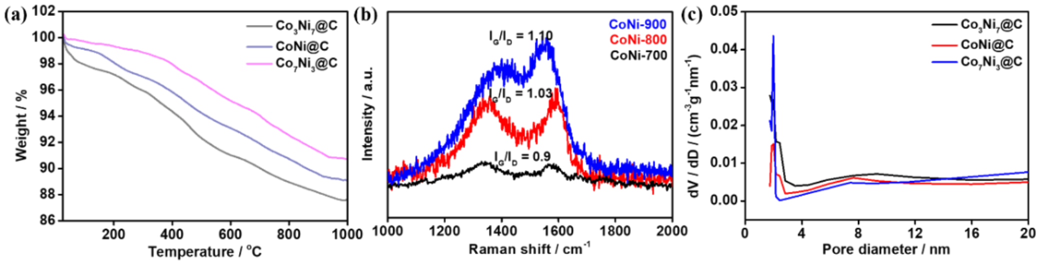

Fig. S2 a TG curves of Co_xNi_y@C composites. **b** Raman spectra of CoNi@C nanosheets with different heating treatment temperature. **c** pore size distribution plots of Co_xNi_y@C composites

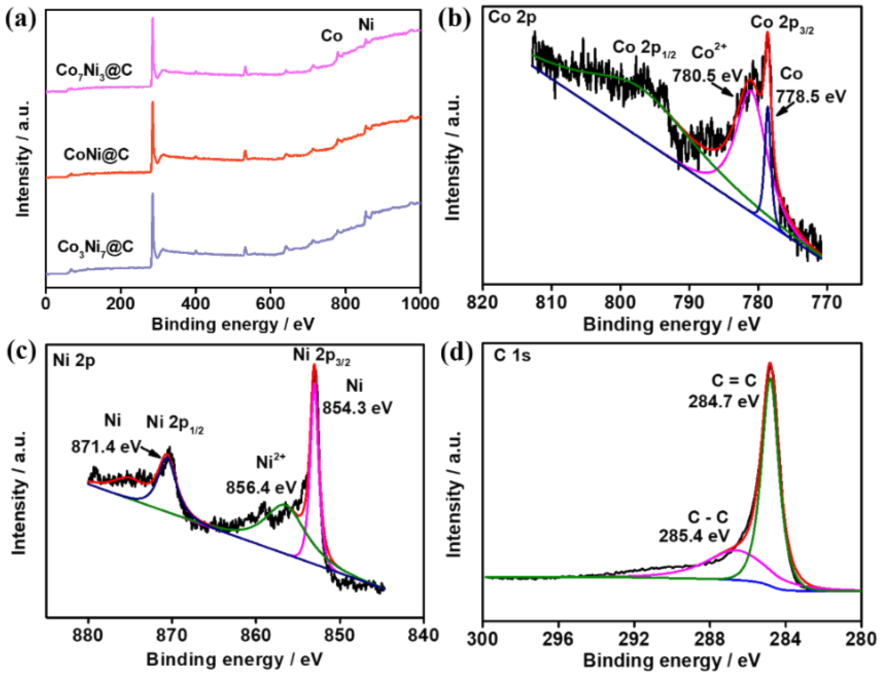

Fig. S3 a XPS survey of Co_xNi_y@C nanosheets. **b** Co 2p, **c** Ni 2p and **d** C 1s XPS survey of CoNi@C nanosheets

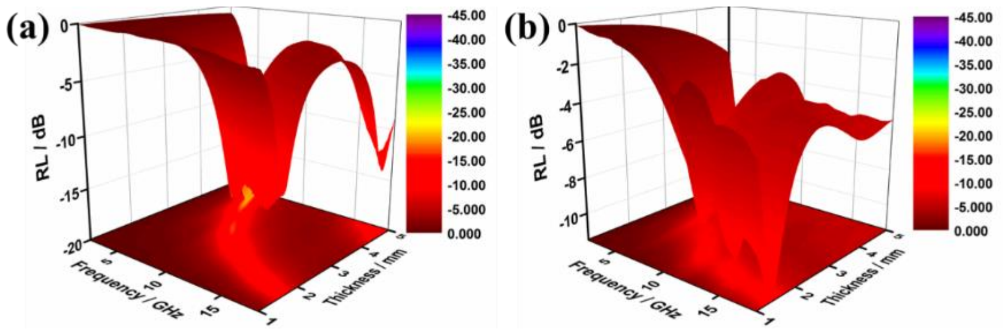

Fig. S4 3D RL plots of **a** Co_3Ni_7@C and **b** Co_7Ni_3@C nanosheets

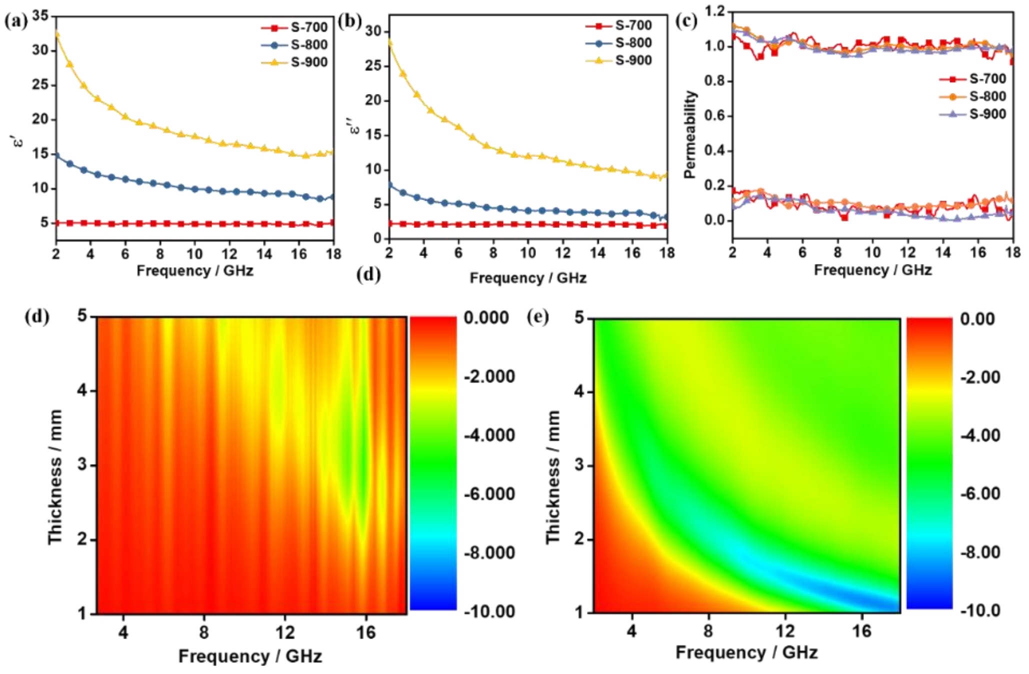

Fig S5 a Real and **b** imaginary part of permittivity and **c** permeability with different temperature. RL of **d** S-700 and **e** S-900

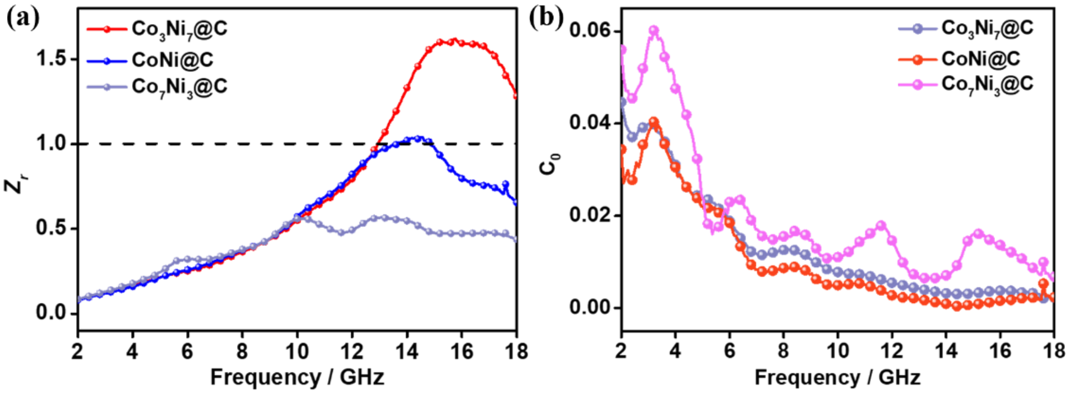

Fig. S6 a Z_r and **b** C_0 values of Co_xNi_y@C composites

Table S1 Microwave absorption property of the similar reports and this work

	RL (dB)	Thickness (mm)	Filler loading ratio (wt%)	Refs.
Ni/Al_2O_3/CNCs (Carbon nanocoil)	-40.3	3.1	25	[1]
CNT/PANI	-47.66	2	25	[2]
HPC (Hierarchically porous carbon)	-62.2	2.71	30	[12]
CoNi-C	-50.2	4	50	[18]
FeCo@C	-21.7	1.2	50	[24]
$Co_{1-x}S$	-46.1	2.5	30	[34]
RBC (Red blood cell like carbon)	-36.6	2	10	[35]
Fe_7Co_3/ZnO	-21.99	2.3	93	[9]
Co_xNi_y/C	-35	2.2	50	[10]
ZnO/Fe/Fe_3C	-30.4	1.5	40	[13]
TiO_2/RGO/Fe_2O_3	-44.05	2	60	[16]
MoC/Co@C	-47.98	1.6	35	[17]
CoNi@C	-43.7	1.7	20	This work
